# Artificial Intelligence in Electrocardiography: From Automated Arrhythmia Detection to Predicting Hidden Cardiovascular Disease

**DOI:** 10.7759/cureus.94065

**Published:** 2025-10-07

**Authors:** Ramy Elantary, Samar Othman

**Affiliations:** 1 Acute Medicine, Mersey and West Lancashire Teaching Hospitals NHS Trust, Merseyside, GBR

**Keywords:** ِartificial intellegence, cardiology, common cardiovascular presentations, electrocardiography (ecg), technology development

## Abstract

Cardiovascular diseases are among the most prevalent and deadly diseases affecting humans. The most widely used diagnostic tool to interrogate cardiovascular physiology and function is an electrocardiogram (ECG). Despite its widespread availability and use, the ECG is subject to interobserver variability and suboptimal sensitivity for asymptomatic or early-stage disease. Artificial intelligence (AI), particularly deep learning (DL) approaches, has provided a suite of methods to improve both the diagnostic and prognostic utility of the ECG in multiple cardiovascular domains. AI-enabled automated ECG interpretation (most commonly using convolutional neural networks (CNNs)) has reached and even surpassed expert-level performance for arrhythmia detection and classification. Additional data-driven approaches to ECG analysis have identified paroxysmal atrial fibrillation from a record of sinus rhythm ECGs, identified left ventricular systolic dysfunction, and predicted cardiac structure and ischemic burden (e.g., acute coronary syndromes). Pragmatic implementation has demonstrated higher diagnostic yield for asymptomatic left ventricular dysfunction in the primary care setting (EAGLE). Other emerging indications include expanded data-derived outputs, such as electrolyte disturbances, biological age, and cardiovascular risk prediction. Despite a growing list of promising applications, numerous translational hurdles remain before routine implementation. Generalizability is limited due to differences in training and target populations. Bias related to sex, race, and comorbidities is an important limiting factor to fair and equitable implementation. Other considerations include “black box” concerns with DL, clinical interpretability and adoption, medicolegal liability, and integration with clinical workflows and infrastructure. Related to these factors, data privacy, algorithmic fairness, accountability, and transparency are important to consider as AI-ECG continues to undergo regulatory scrutiny and outcomes-based validation. In conclusion, AI and ECG represent a major shift towards precision cardiology by improving prediction, screening, and early detection of cardiovascular disease. We anticipate continued improvements with prospective outcome studies, transparent and explainable approaches, and careful regulatory review to ensure safe and effective implementation in the clinic.

## Introduction and background

An electrocardiogram (ECG) has been a mainstay in cardiovascular diagnostics for over a century since Willem Einthoven's integration into clinical medicine in 1908 [1]. As a noninvasive, low-cost, and readily available modality, the ECG yields important information about cardiac rhythm, conduction, and ischemia and remains a key guide for clinical decision-making in both acute and chronic settings [2]. Conventional ECG interpretation remains constrained by its reliance on clinician expertise, with diagnostic accuracy and reproducibility often affected by interobserver variability, particularly in the evaluation of subtle repolarization abnormalities or nonspecific ST-T wave changes [3]. Automated rule-based algorithms present in many ECG machines have been widely adopted; however, their diagnostic performance often remains inferior to expert interpretation, with reported sensitivities ranging from 70% to 85% and specificities from 80% to 90%, depending on the condition and algorithm used, as will be discussed subsequently [4].

Artificial intelligence (AI) and machine learning (ML) are powerful analytical tools that can detect hidden patterns in large, complex datasets, and their evolution has advanced swiftly over the past decade. Cardiology, particularly the ECG, has been an area of growing interest for AI development due to its widespread adoption, digital nature, and rich data content [5]. DL models such as convolutional neural networks (CNNs) take advantage of these analytical capabilities, where models can ingest raw waveform data and perform increasingly abstract feature engineering on their own, without direct human manipulation, to then be used for pattern recognition at higher levels beyond human visual comprehension [6]. These systems showed initial proof-of-concept by learning to accurately classify arrhythmias from single-lead ECGs in a manner that was on par with board-certified cardiologists [7].

Applications of AI-based ECG analysis have only grown since. AI-ECG models were subsequently found to be able to predict left ventricular systolic dysfunction from sinus rhythm ECGs [8], detect evidence of prior atrial fibrillation even in patients in normal sinus rhythm [9], and even for screening of metabolic abnormalities such as hyperkalemia [10]. These models have also been shown to be clinically useful. Pragmatic trials like the EAGLE study have demonstrated that screening for low ejection fraction using AI-ECG increased detection in primary care settings beyond what had been achieved previously [11]. In 2023, the first AI-ECG algorithm was approved by the United States Food and Drug Administration for the detection of low ejection fraction [12], a landmark event of the first AI algorithm in cardiology to transition from research setting to clinical use.

However, important gaps remain. The majority of these models are “black boxes” with limited interpretability, which impairs trust and medicolegal responsibility [13]. Model performance is also frequently population-dependent, which could lead to sex-, race-, or comorbidity-related bias. For example, deep learning (DL) models trained predominantly on data from middle-aged male populations may underperform when applied to females, older adults, or individuals with differing comorbidity profiles, leading to reduced diagnostic accuracy in these groups [14]. Integration of these tools into clinical workflows, health record systems, and reimbursement infrastructure is also very limited. Solving these challenges will be required for broad adoption.

Taken together, the application of AI to ECG interpretation has the potential to shift paradigms in cardiovascular medicine by introducing greater diagnostic consistency and redundancy, thereby enhancing system reliability. Hidden signals within a century-old technology have the potential to improve screening, risk prediction, and clinical decision-making on an individualized basis. The next section will discuss the approaches taken towards AI-ECG development, including the sources and use of data, the common architectures of models, validation strategies, and emerging methods to improve transparency and generalizability.

Methodology

This narrative review of the literature was not intended to be a systematic review with meta-analysis of selected research studies, but instead to focus on the breadth of coverage and clinical relevance of the information. The authors searched through the available literature to provide the most up-to-date and comprehensive evidence of artificial intelligence (AI)-enabled ECG interpretation, in terms of diagnostic accuracy, clinical utility, and real-world application, as well as approved indications and future directions.

Electronic searches of PubMed/MEDLINE, Embase, Scopus, and Google Scholar were performed to provide peer-reviewed articles and high-impact conference proceedings. The websites of major cardiovascular societies (e.g., American College of Cardiology, European Society of Cardiology, American Heart Association) were also hand-searched to reduce selection bias and to include recent position statements, consensus documents, and guideline updates. ClinicalTrials.gov was screened for active or recently completed trials on the topic of AI-ECG technologies.

Searches were conducted using the following keywords and MeSH terms in combination: “artificial intelligence”, “machine learning”, “deep learning”, “electrocardiogram”, “ECG”, “EKG”, “arrhythmia”, “atrial fibrillation”, “heart failure”, “left ventricular dysfunction”, “myocardial infarction”, “ischemia”, and “electrolyte abnormality”. Boolean operators (AND/OR) were used to combine search terms. Filters were used to restrict search results to those with a focus on human studies and those published in English.

Search dates spanned from January 2015 through August 2025. The search window was chosen because the first landmark reports of DL for ECG analysis were published in 2016-2017, and the present aim was to identify the most important, high-quality work in the modern era of AI. References to older literature were included solely when essential for historical context, such as traditional approaches to ECG interpretation.

A total of 1,420 unique records were identified in the search across databases. After removal of duplicates and application of inclusion/exclusion criteria, 112 full-text articles were screened. Study selection was conducted in two stages: initial screening of titles and abstracts, followed by full-text review of potentially eligible studies.

Studies were included if they involved the application of AI or ML techniques to electrocardiographic data for diagnostic, predictive, or prognostic purposes; reported original human research using standard 12-lead, single-lead, or wearable ECG data; and were published in peer-reviewed journals in English. Both retrospective and prospective designs were accepted, including randomized controlled trials, observational cohorts, and validation studies. Systematic reviews and meta-analyses were considered when they synthesized primary data relevant to AI-ECG applications.

Exclusion criteria included studies that used non-human or simulated ECG data only, lacked a clear description of the AI methodology or validation process, focused exclusively on signal-processing or algorithmic development without clinical interpretation, or presented data solely in preprint or unpublished form. Conference abstracts, opinion pieces, and studies without an accessible full text were also excluded.

A total of 68 studies were included in the present narrative review. This included landmark randomized controlled trials (e.g., EAGLE), key validation studies of arrhythmia and left ventricular dysfunction detection, important observational studies of electrolyte imbalance and myocardial infarction prediction, and relevant position statements or regulatory reports.

Information was abstracted from each study regarding study design, sample size, and population, AI model architecture, primary outcomes, and clinical implications. Results were synthesized narratively according to clinical application area (arrhythmia detection, AF in sinus rhythm, left ventricular dysfunction, MI/ischemia, electrolyte abnormalities, and emerging biomarkers). Emphasis was placed on clinically validated findings and external validation; novel methodological approaches lacking clinical evaluation were not discussed in detail, as the focus of this review is on clinically validated methods.

In summary, this review integrates the evidence from the 2015-2025 literature and includes 68 studies identified through a structured narrative search of the major biomedical literature databases and cardiovascular society resources. This approach allowed for a systematic yet clinically oriented synthesis of the rapidly evolving field of AI-enabled ECG interpretation.

## Review

Clinical applications of AI in ECG

Application areas of AI to electrocardiography have now broadened from traditional rhythm analysis to structural, metabolic, and prognostic categories. In all six application categories of AI-ECG reviewed here (arrhythmia detection, atrial fibrillation during sinus rhythm, left ventricular dysfunction, myocardial infarction, electrolyte abnormalities, and emerging uses), diagnostic accuracy has been shown to be at least as good as expert interpretation.

AI models perform these tasks quite differently from human readers. While a clinician detects disease through familiar wave morphology, the AI model treats the ECG as high-dimensional time-series data. Using CNNs, the algorithm detects and learns reproducible micro-patterns in the waveforms - patterns that are invisible to the human eye but are associated with disease phenotypes. In atrial fibrillation prediction during sinus rhythm, the model discerns slight variations in atrial conduction that will lead to arrhythmia; in heart failure, the algorithm learns the dispersed ventricular activation and repolarization that predicts low ejection fraction; and for electrolyte abnormalities, it detects amplitude and slope features of T-waves with far greater sensitivity than rule-based approaches. Training on millions of examples, the network internalizes these latent “signatures” of disease without explicit rules.

This ability to reveal hidden signals endows AI-ECG with the potential to be both diagnostic and predictive, as a biomarker. The unifying feature across these applications is that the technology detects subclinical disease, i.e., disease that is below the threshold of human detection. The implication is that a 100-year-old, low-cost test can be repurposed as a predictive screening platform that will enable earlier diagnosis and risk stratification at scale, as well as preventive interventions.

Arrhythmia Detection and Classification

AI's ability to classify arrhythmias was convincingly demonstrated in a retrospective observational study by Hannun et al. (2019), which examined over 91,000 single-lead ambulatory ECGs [8]. A deep neural network was trained on 12 rhythm classes in this work, and its performance on an independent test set was cardiologist-level. This paper's significance was that it showed that AI could distil diagnostic information from raw electrocardiographic waveforms at a level that matched or outperformed human experts.

The CODE study by Ribeiro et al. (2020) was a later study that instead used over two million 12-lead ECGs gathered from a national Brazilian telehealth system [14]. This was also a retrospective observational study, but its DL algorithm was shown to outperform cardiology residents in six common electrocardiographic abnormalities, demonstrating AI's feasibility for population-scale arrhythmia screening.

In a parallel effort, the PhysioNet Computing in Cardiology Challenge (2017) released a large open annotated set of single-lead tracings for the classification of atrial fibrillation and other rhythms, creating a standard benchmarking platform [15]. Despite the competition’s non-clinical setting, standardization of evaluation metrics and a common open testing platform enabled global crowdsourcing of methodological innovation and development, jump-starting the field.

Translation to clinical practice was demonstrated by the Apple Heart Study (2019), a pragmatic prospective trial enrolling more than 419,000 smartwatch users [16]. In this algorithmic assessment, an irregular pulse notification algorithm triaged to ambulatory electrocardiographic patch monitoring, and a third of notified participants were subsequently confirmed to have atrial fibrillation. The positive predictive value of concurrent notification and patch-confirmed atrial fibrillation was 0.84, showing the potential for population-level, AI-assisted, real-world rhythm surveillance at scale.

Together, these studies indicate that AI can match or exceed human experts in arrhythmia detection. These results are summarized in Table 1, which outlines the major studies, their design, and principal findings.

**Table 1:** Key studies on artificial intelligence-based electrocardiogram (AI-ECG) for arrythmia detection and classification Created by the authors

Study	Year	Type of study	Population / dataset	Main findings
Hannun et al. [8]	2019	Retrospective observational	91,232 single-lead ambulatory ECGs	Deep neural network classified 12 arrhythmias with cardiologist-level accuracy.
Ribeiro et al. (CODE) [14]	2020	Retrospective observational	>2 million 12-lead ECGs (Brazil)	Outperformed cardiology residents in detecting six common abnormalities.
PhysioNet CinC Challenge [15]	2017	International benchmarking study	Open annotated single-lead ECG data	Standardized evaluation framework for atrial fibrillation and other rhythms.
Apple Heart Study [16]	2019	Pragmatic prospective trial	≈419,000 smartwatch users	34% of irregular pulse notifications confirmed AF on the patch; PPV for AF = 0.84.

Detection of Atrial Fibrillation During Sinus Rhythm

In Attia et al.'s study, a DL algorithm was trained on 180,000 plus 12-lead ECGs at the Mayo Clinic to predict patients with prior atrial fibrillation when the index 12-lead ECG was in sinus rhythm [17]. The algorithm performed well with an area under the curve of 0.87. This retrospective observational study at a single institution suggested that there are latent electrical signatures of atrial fibrillation that are undetectable by human interpretation.

Raghunath et al. demonstrated this idea in a large retrospective multi-center validation study across multiple health systems on more than 430,000 patients [18]. Using deep neural networks, their model predicted new-onset atrial fibrillation in the one year after a sinus rhythm ECG with superior performance to traditional clinical risk scores. These data showed that AI is able to risk-stratify patients for early monitoring and preventive anticoagulation.

The clinical relevance of these algorithms was confirmed by Khurshid et al. in a prospective observational study at Massachusetts General Hospital [19]. Patients deemed to be high risk by an AI-ECG algorithm experienced higher rates of device-detected atrial arrhythmias over follow-up.

Taken together, these studies suggest that AI algorithms can detect latent signals of atrial fibrillation risk on routine sinus rhythm ECGs, providing a new opportunity for early detection and preventive management in patients with cryptogenic stroke.

An overview of the principal studies evaluating this application is presented in Table 2.

**Table 2:** Key studies on artificial intelligence-based electrocardiogram (AI-ECG) for atrial fibrillation detection during sinus rhythm Created by the authors

Study	Year	Type of study	Population / dataset	Main findings
Attia et al. [17]	2019	Retrospective observational	>180,000 ECGs, Mayo Clinic	Identified prior AF from sinus rhythm with AUC 0.87.
Raghunath et al. [18]	2021	Retrospective multi-center	430,000+ patients, several US health systems	Predicted new-onset AF within 1 year, outperforming clinical risk scores.
Khurshid et al. [19]	2022	Prospective observational	MGH cohort, device monitoring follow-up	AI-ECG high-risk patients had more device-detected atrial arrhythmias.

*Prediction* *of* *Left* *Ventricular* *Dysfunction* *and* *Heart* *Failure*

Attia et al.'s study (2019) was a retrospective, observational study at the Mayo Clinic, in which a DL algorithm was trained on over 44,000 12-lead ECGs associated with echocardiographic data [5]. The model was able to identify patients with left ventricular ejection fraction below 35% from sinus rhythm tracings with an area under the curve of 0.93. It was one of the first studies to demonstrate the ability of AI to recognize ventricular dysfunction without imaging using routine electrocardiography alone.

The EAGLE trial (2021) was a pragmatic, randomized controlled trial performed in primary care practices throughout Minnesota and Wisconsin [10]. In this pragmatic trial, more than 22,000 patients were screened using an AI-enabled ECG algorithm. The researchers found that patients who were randomized to AI-guided screening were significantly more likely to receive a new diagnosis of low ejection fraction within three months as compared with usual care. It provided the first prospective evidence that AI-ECG tools can improve the detection of heart failure in the real-world clinical setting.

Subsequently, Jentes et al.'s study (2022) was a retrospective, multi-center study that analyzed over 100,000 ECGs from European cohorts [20]. The AI-ECG maintained high levels of accuracy in diverse health systems, demonstrating the generalizability of this technology.

Collectively, these studies indicate that AI-ECG can be a cost-effective, scalable screening tool for ventricular dysfunction, which could support the earlier diagnosis of heart failure and reduce dependence on resource-intensive echocardiography.

The most relevant studies and their clinical implications are summarized in Table 3.

**Table 3:** Key studies on artificial intelligence-based electrocardiogram (AI-ECG) for the prediction of left ventricular dysfunction and heart failure Created by the authors

Study	Year	Type of study	Population / dataset	Main findings
Attia et al. [5]	2019	Retrospective observational	44,000+ ECGs, Mayo Clinic	Detected low EF (<35%) from sinus rhythm with AUC 0.93.
EAGLE trial [10]	2021	Pragmatic randomized trial	22,000+ primary care patients	AI-ECG arm had a higher rate of new EF <50% diagnoses within three months vs. usual care.
Jentes et al. [20]	2022	Retrospective multi-center	100,000+ ECGs, European cohorts	AI-ECG for low EF showed strong external validity across diverse health systems.

*Detection* of *Myocardial* *Ischaemia* *and* *Infarction*

Herman et al.'s study (2023) was a retrospective, international, multi-center diagnostic evaluation of an AI-ECG model trained to detect occlusion myocardial infarction [21]. The investigators compared the algorithm to conventional ST-segment elevation criteria and expert rules across heterogeneous health systems. The AI model was more sensitive for identifying angiographically proven coronary occlusion, including cases without classic ST-segment elevation, and did so with clinically acceptable specificity. In practical terms, this work suggested that AI could reduce time to reperfusion by flagging patients who would be missed or delayed by standard criteria, thus improving triage decisions in the emergency department and pre-catheterization laboratory pathways.

Strodthoff and Wagner (2020) published the PTB-XL resource and benchmarking study that contributed a large, publicly available, 12-lead ECG database with strong labeling, including myocardial infarction categories [22]. While not a clinical trial, this data set established a foundation for reproducible method development: multiple DL models trained on PTB-XL showed strong discrimination of myocardial infarction and non-infarction classes on retrospective testing, allowing for transparent head-to-head comparison and fast external replication. The curation effort and standard splits also enabled more reliable reporting of diagnostic accuracy as well as subsequent research into generalizability across institutions and vendors.

The international diagnostic evaluation and the standardized benchmarking resource together provide a window into the evolution of AI-ECG tools for myocardial infarction from retrospective proof-of-concept toward deployment-ready decision support. The near-term use case most likely to see clinical adoption is AI triage to augment clinician interpretation: fast flagging of possible occlusion myocardial infarction to expedite confirmatory testing and reperfusion decision-making.

However, beyond their diagnostic accuracy, it is essential to consider the real-world implications of these algorithms, particularly the balance between sensitivity and false-positive alerts. While retrospective benchmarking using large annotated datasets such as PTB-XL has demonstrated remarkable diagnostic accuracy for ischemic ECG changes, the translation of these results into clinical settings remains complex. False positives are a particular concern: when AI systems flag benign or nonspecific repolarization patterns as ischemic, they can trigger unnecessary testing, resource utilization, and patient anxiety. In Herman et al.'s multicenter evaluation, although sensitivity was high, the rate of false positives varied between 5% and 15% depending on the clinical threshold applied. Such findings illustrate the trade-off between maximizing sensitivity for life-threatening events and maintaining specificity to avoid alert fatigue and downstream burden. Integrating AI-ECG outputs into structured triage protocols or multi-modal decision systems-combining ECG data with clinical features and biomarkers-may mitigate this issue. Continued prospective evaluation is essential to quantify the clinical and economic implications of false positives before large-scale deployment.

The main studies with this regard and their main findings are detailed in Table 4.

**Table 4:** Key studies on artificial intelligence-based electrocardiogram (AI-ECG) for myocardial ischemia and infarction Created by the authors

Study	Year	Type of study	Population / dataset	Main findings
Herman et al. [21]	2023	Retrospective international multi-center evaluation	Emergency and hospital electrocardiograms across sites	Higher sensitivity than conventional ST-segment elevation criteria for occlusion myocardial infarction with acceptable specificity; supports faster triage to reperfusion.
Strodthoff and Wagner (PTB-XL) [22]	2020	Public dataset and benchmarking study	Large 12-lead electrocardiogram database with labels	Enabled reproducible training and comparison of models; retrospective models showed strong discrimination of myocardial infarction versus non-infarction classes.

Electrolyte Abnormalities and Systemic Disorders

Although DL models for detecting electrolyte disturbances, particularly hyperkalemia, have shown strong diagnostic performance, most remain in the investigational or pre-regulatory phase.

In a retrospective observational study by Galloway et al. (2019), over 1.5 million patients' paired serum potassium and ECGs from a large United States health system were used [9]. A DL model was trained to predict hyperkalemia directly from 12-lead ECGs. The resulting algorithm achieved high accuracy with a negative predictive value greater than 95%, demonstrating that a normal AI-ECG can be used to effectively rule out clinically significant hyperkalemia. This algorithmic performance has potential triage applications for use in emergency departments and dialysis units when laboratory assessment of electrolyte derangements is often delayed.

This was expanded by Harmon et al. (2024) in a prospective validation study in multiple nephrology clinics [23]. Patients receiving routine hemodialysis were evaluated by simultaneous serum potassium testing and AI-ECG analysis. As in previous studies, the model was found to have high concordance with laboratory values and, more importantly, could successfully identify severe hyperkalemia that necessitated emergent treatment. The prospective design helped to alleviate any remaining doubt regarding the pragmatic implementation of AI-enabled ECG analysis in chronic kidney disease care.

Hyperkalemia has not been the only systemic abnormality that has been the focus of AI model creation. Hypocalcemia, anemia, and thyroid dysfunction are other examples of conditions that have been successfully identified by AI algorithms using routine ECGs, but these studies remain largely retrospective and exploratory, and these algorithms are not yet available for clinical use [24].

Electrolyte detection is one use case for AI-ECGs that is practical, clinically actionable, and relevant for high-risk populations. One example would be the detection of electrolytes in chronic kidney disease patients, where hyperkalemia is best validated, but other systemic disorders may be identifiable with larger models and datasets.

A summary of these studies and the findings is provided in Table 5.

**Table 5:** Key studies on artificial intelligence-based electrocardiogram (AI-ECG) for electrolyte abnormalities and systemic disorders Created by the authors

Study	Year	Type of study	Population / dataset	Main findings
Galloway et al. [9]	2019	Retrospective observational	1.5M+ ECGs linked with serum potassium levels	AI-ECG predicted hyperkalemia with high accuracy; negative predictive value >95%.
Harmon et al. [23]	2024	Prospective validation	Hemodialysis patients in nephrology clinics	AI-ECG closely matched lab potassium; effective in identifying severe hyperkalemia.
Exploratory reports [24]	2020–2023	Retrospective pilot studies	Various hospital datasets	Suggested feasibility for detecting hypocalcemia, anemia, thyroid dysfunction; not yet validated.

Emerging and Novel Applications of AI-ECG

Ko et al. (2021) used a retrospective observational cohort study using the UK Biobank dataset to train AI models on >500,000 ECGs [25]. They showed that a neural network can predict chronological age and biological sex directly from the ECG with very high accuracy. More importantly, patients with a higher “electrocardiographic age” than their chronological age had a significantly higher risk of all-cause and cardiovascular mortality, suggesting that the AI-enabled ECG can serve as a novel biomarker of biological aging and long-term risk stratification.

In the field of structural heart disease, Attia et al. (2022) used a retrospective study that integrated ECG data with echocardiographic outcomes to develop an AI-ECG algorithm for detecting hypertrophic cardiomyopathy and other structural abnormalities [26]. This ECG algorithm demonstrated promising accuracy in detecting these conditions. These results highlight the potential of ECGs to transform from a tool for rhythm and conduction to one for structural screening. This is particularly relevant in low-resource settings, where ECGs are more accessible than echocardiography.

Finally, Poterucha et al. (2025) recently reported a prospective proof-of-concept study called EchoNext that prospectively tested multimodal AI models built using ECGs, electronic health record (EHR)-derived features, and echocardiographic data [27]. This study demonstrated that the integration of the AI-enabled ECG with other data sources can further improve predictive performance, bringing the field one step closer to precision cardiology.

Novel use cases such as biological age prediction, structural heart disease detection, and multimodal integration highlight the versatility of the AI-ECG. Although most are in early stages, they suggest that the ECG could become a more general tool for predicting cardiovascular health beyond diagnosis.

These models - focused on predicting physiological age, detecting structural heart disease, and integrating multimodal data with imaging or clinical records - are presently in early investigational or translational phases. None of these systems has yet obtained formal regulatory approval from the U.S. Food and Drug Administration (FDA) or comparable authorities. Although the mentioned algorithms have shown high discriminative performance and strong potential for population-level screening, they require prospective, multicenter validation and demonstration of clinical utility before routine implementation can be justified.

The mentioned principal contributions in this area are summarized in Table 6.

**Table 6:** Key studies on emerging and novel applications of artificial intelligence-based electrocardiogram (AI-ECG) Created by the authors

Study	Year	Type of study	Population / dataset	Main findings
Ko et al. [25]	2021	Retrospective observational	500,000+ ECGs, UK Biobank	Predicted age/sex; discrepancy between ECG age and true age predicted higher mortality.
Attia et al. [26]	2022	Retrospective observational	ECG + echocardiographic cohorts	Detected hypertrophic cardiomyopathy and other structural abnormalities with good accuracy.
Poterucha et al. [27]	2025	Prospective proof-of-concept	EchoNext multimodal study	Integrated ECG with EHR and imaging; improved predictive power for cardiovascular outcomes.

Challenges and future directions

As the field of AI-ECG technology has matured, increasingly complex diagnostic tasks are being approached with an accuracy that was once thought impossible with only a surface tracing. With these rapid advances in diagnostic accuracy come obstacles and opportunities that may define the space of cardiovascular AI-ECG over the coming years. Barriers include generalizability, equity, interpretability, workflow integration, regulatory and legal considerations, and a need for outcomes-driven evidence. This is true in parallel to several other expanding frontiers that have great potential to guide future research and clinical translation.

*Generalizability* *and* *External* *Validation*

One area of continued concern in AI-ECG is the question of whether or not a model trained in one population can be applied to different populations with the same performance. The majority of the seminal algorithms, including those for low ejection fraction or atrial fibrillation prediction, have been trained in a single center with a mostly homogenous population. Performance at external testing populations has often been shown to suffer. Karabayir et al. (2025) performed a systematic review of validation studies of AI-ECG algorithms across multiple health systems and found that while the majority of algorithms were able to provide some degree of prediction, accuracy was often dependent on patient-level factors, such as demographics and comorbidity burden, and even technical factors such as ECG acquisition hardware [28]. In a similar line of work, Jentes et al. (2022) tested Attia’s ejection fraction algorithm on European cohorts and found generalization with modest reductions in sensitivity as compared with the original Mayo Clinic cohort reports [20]. These and other related studies point to the critical importance of multi-institutional, diverse training datasets and the need for rigorous, standardized external validation protocols.

*Bias* *and* *Equity*

Tightly linked to the concept of generalizability is the issue of bias. For example, if AI-ECG models have been trained with datasets that poorly represent historically disadvantaged groups, these models can perpetuate existing inequalities. Mihn et al. (2024) recently showed that the diagnostic performance of AI-ECG systems is variable by sex and race, with significantly increased false negative rates in women and minority populations in the detection of arrhythmias [29]. Commentaries in Circulation have since argued that AI has the potential to exacerbate existing disparities in cardiovascular outcomes if these biases are not systematically corrected [29]. Moving forward, this will require inclusive data curation, reporting on subgroup performance, and the development of fairness-aware algorithms. 

*Interpretability* *and* *Clinician* *Trust*

In addition to generalizability concerns, many clinicians are understandably cautious to accept recommendations from “black box” models. The opaque nature of these algorithms can erode trust and create liability concerns. A recent review by Siontis et al. (2021) explored explainability approaches such as saliency maps and attention heatmaps in the setting of AI-ECG and found that these tools can offer some insight into which features of the waveform are responsible for predictions, but are often inconsistent and may not correlate with clinical understanding [30]. Robust standards for model interpretability will be required for these tools to be widely adopted. This is a challenge that goes beyond cardiology, but one that is especially relevant when high-stakes treatment decisions are at stake.

Integration Into Clinical Workflow

Another issue is that even if performance is acceptable on validation studies, deployment of algorithms in clinical practice is nontrivial. The EAGLE trial (2021) found that AI-ECG screening can increase the number of new reduced ejection fraction diagnoses [10]. The Apple Heart Study (2019) confirmed that it is possible to notify users of irregular pulses at scale, but also demonstrated significant management burden with a high number of alerts with limited positive predictive value (PPV) requiring confirmatory tests [16]. Integration of AI-ECG into workflows needs to be seamless to succeed. In addition to presenting results in EHR systems with intuitive visualizations, this includes providing guidance for appropriate actions to reduce alert fatigue.

Legal and Ethical Issues

There is a recent flurry of regulatory activity as well. A major landmark was the first U.S. Food and Drug Administration clearance of an AI-ECG in 2023 (Anumana’s algorithm for detection of low ejection fraction) [11]. This clears the way for AI-ECG to become a widely accepted clinical device, although the medicolegal implications of errors in classification are not yet clear. A recent expert editorial in JACC (2024) warned that liability models for false-negative or false-positive diagnoses using AI are undefined and could have significant implications for both clinicians and institutions [31]. The field is likely to see an increasing number of approved devices, and this should spur the development of harmonized international standards and adaptive approval pathways to balance safety and innovation.

In addition to the aforementioned themes, it is critical that the ethical implications of implementing AI-ECG systems are also made clear and transparent to clinicians. This includes considerations around data privacy and informed consent, the potential for algorithmic bias and subsequent disparate care, and who is held accountable in the event of diagnostic error. Transparency in data provenance and model decision-making is also necessary to sustain clinician and patient trust.

*Clinical* *Utility* *and* *Outcomes* *Evidence*

A major limitation of the current literature is the lack of data on clinical outcomes. Despite dozens of studies showing high diagnostic accuracy, very few have sought to determine if AI-ECG improves on hard outcomes like mortality, hospitalization, or cost-effectiveness. The EAGLE trial (2021) is the first randomized trial to demonstrate improved diagnostic yield, but it did not measure downstream outcomes like hospitalization for heart failure or survival [23]. A recent editorial in NEJM Evidence (2024) highlighted that the evidence base for clinical utility is too limited to draw firm conclusions, and better outcomes data is urgently needed [32]. The field needs to move beyond diagnostic metrics to pragmatic trials powered for patient outcomes.

Economic and cost-effectiveness considerations

The economic potential of AI-ECG, while appreciated, has not been well quantified. Modeling data indicate that AI-enhanced ECG screening could be cost-effective for triaging patients to advanced imaging or focused cardiac evaluation, especially in primary-care or resource-limited settings. This technology would leverage a widely available, low-cost screening tool to improve and potentially decrease downstream testing and treatment costs. The initial investments to AI-enable ECG interpretation in a clinical setting include software architecture, algorithms, and licensing, as well as physician training costs, which may preclude immediate savings. The long-term cost-effectiveness of AI-ECG technology will be driven by the ability to decrease hospitalizations, avoid unnecessary imaging, and reduce cardiovascular events, which has not been validated in large prospective studies.

*Expanding* *Horizons*

Despite these challenges, AI-ECG remains a fast-evolving field with clear future directions. Poterucha et al. (2025) have shown, in the context of the EchoNext study, that multimodal models that incorporated ECG in addition to echocardiography and EHR data far outperformed ECG-only models for the prediction of structural disease [27]. These studies, among others, show a tantalizing glimpse into a future where AI-ECG transcends its current clinical role as a diagnostic test and becomes a tool for precision and even preventative cardiology as a low-cost entry point to the field.

In conclusion, AI-ECG finds itself at an important crossroads. At this point, the research literature points to very promising performance in tasks ranging from arrhythmia detection to heart failure screening, myocardial infarction triage, and even systemic disease prediction. Simultaneously, key challenges in generalizability, bias, interpretability, workflow integration, regulation, and evidence of outcomes impact remain to be resolved before broad clinical adoption can be realized. These will need to be the foci of next-generation research, which must aim to ensure external validation, data curation diversity and transparency, interpretability that is actionable in a clinical workflow, and integration into trials that are truly focused on patient outcomes. If these challenges can be met, AI-ECG could revolutionize a diagnostic modality that has been used in its current form for over a century.

## Conclusions

AI for ECG has seen a swift translation from proof-of-concept studies to clinical applicability. In the realms of arrhythmia recognition, anticipation of atrial fibrillation in sinus rhythm, detection of left ventricular dysfunction, ischemia, and electrolyte abnormalities, AI-ECG models have shown diagnostic performance on par with or exceeding that of human experts. These systems not only replicate human-level performance but also extract patterns from ECG waveforms that are not always apparent to human observers. These patterns can reveal a preclinical disease state or indicate risk before the disease becomes clinically manifest. As such, the ability to repurpose a low-cost, widely available, noninvasive test into a form of disease biomarker with prognostic value is a promising innovation in cardiology. Applications are beginning to appear that may enable structural disease screening, biological age determination, and multimodal fusion with cardiac imaging and electronic health record data. These innovations are rapidly pushing the limits of traditional electrocardiography, positioning AI-ECG as a potential tool for not just diagnosis, but also for population screening, triage, and preventive medicine.

Simultaneously, careful consideration is necessary for translation to clinical routine. Generalizability to different populations and health systems, elimination of algorithmic bias against underrepresented populations, interpretability to clinician users, integration into electronic health record systems to avoid alert fatigue and workflow disruption, and regulatory and medicolegal responsibility for misclassification are only beginning to be clarified. Crucially, beyond diagnostic accuracy, evidence from outcomes-driven trials will be needed to demonstrate that deployment of AI on the ECG actually improves survival, reduces hospitalizations, and results in cost-effective care. If these challenges can be overcome with solid evidence and careful implementation, AI-ECG has the potential to reinvent this 100-year-old tool as a flexible platform for predictive, preventive, and personalized cardiology beyond the specialist clinic to primary care and global health.
